# Foreign Body in the Urethra

**Published:** 1883-11

**Authors:** 


FOREIGN BODY IN THE URETHA.—DR. GEORGE HUNTER, M.D., Linlithgow, writes:

"An elderly gentleman, the subject of dysuria from prostatic enlargement, thought to aid the efforts of his bladder in its evacuation by insinuating the rounded head of his wife's veil-pin into the orifice of his urethra, and thereby opening up the passage. To his dismay, in its descent downwards it slipped from his fingers, and the point of the pin disappeared from his sight. His attempts at removal only caused it to make its way further back, and soon a discharge of mucus, and urgent but ineffectual attempts to pass urine, alarmed him, and induced him to send for me. On my arrival, I could just make out the head of the pin in the membranous urethra in front of the prostate, and could feel the point anterior to the scrotum. To remove it, I fixed the head by pressing on it from behind forwards, and then impaled the urethra against the point. By steady pressure and traction on the point as soon as it emerged from the under surface of the penis, the whole length of the pin was pulled through, only the head remaining in the urethra. The point was then depressed towards the perinæum, and by compressing the flaccid penis in its longitudinal axis, the round head of the pin was easily passed through the meatus, and the entire pin withdrawn. In its removal not a drop of blood was lost, and the puncture remaining was not more severe than that resulting from the use of the ordinary hypodermic needle. Beyond enjoining rest and quiet for the first twelve hours, nothing further was prescribed, and my patient was next day in his usual health."—*British Medical Journal.*

Under date of September 12th, 1883, the *London Medical Press and Circular* informs its readers that the first of certain proposed "retiring rooms for females" has been opened in Gray's Inn Road. We cannot but feel wonder as to what sort of *females* are about to retire to such places. Is it, for example, the female of the British Lion who is thus favored? Perhaps, O, yes! perhaps it is the *women* of London who are to be thus accommodated! But why do not our English friends call their sisters by their honest English name?

